# Maintenance of influenza A viruses and antibody response in mallards (*Anas platyrhynchos*) sampled during the non-breeding season in Alaska

**DOI:** 10.1371/journal.pone.0183505

**Published:** 2017-08-24

**Authors:** Timothy J. Spivey, Mark S. Lindberg, Brandt W. Meixell, Kyle R. Smith, Wendy B. Puryear, Kimberly R. Davis, Jonathan A. Runstadler, David E. Stallknecht, Andrew M. Ramey

**Affiliations:** 1 Department of Biology and Wildlife, University of Alaska Fairbanks, Fairbanks, Alaska, United States of America; 2 U.S. Geological Survey, Alaska Science Center, Anchorage, Alaska, United States of America; 3 Institute of Arctic Biology, University of Alaska, Fairbanks, Alaska, United States of America; 4 Alaska Department of Fish and Game, Anchorage, Alaska, United States of America; 5 Department of Biological Engineering & Division of Comparative Medicine, Massachusetts Institute of Technology, Cambridge, Massachusetts, United States of America; 6 College of Veterinary Medicine, Department of Population Health, University of Georgia, Athens, Georgia, United States of America; The University of Chicago, UNITED STATES

## Abstract

Prevalence of influenza A virus (IAV) infections in northern-breeding waterfowl has previously been reported to reach an annual peak during late summer or autumn; however, little is known about IAV infection dynamics in waterfowl populations persisting at high-latitude regions such as Alaska, during winter. We captured mallards (*Anas platyrhynchos*) throughout the non-breeding season (August–April) of 2012–2015 in Fairbanks and Anchorage, the two largest cities in Alaska, to assess patterns of IAV infection and antibody production using molecular methods and a standard serologic assay. In addition, we used virus isolation, genetic sequencing, and a virus microneutralization assay to characterize viral subtypes and to evaluate the immune response of mallards captured on multiple occasions through time. We captured 923 mallards during three successive sampling years: Fairbanks in 2012/13 and 2013/14, and Anchorage in 2014/15. Prevalence varied by age, season, and year/site with high and relatively stable estimates throughout the non-breeding season. Infected birds were detected in all locations/seasons except early-winter in Fairbanks during 2013/14. IAVs with 17 combinations of hemagglutinin (H1–5, H7–9, H11, H12) and neuraminidase (N1–6, N8, N9) subtypes were isolated. Antibodies to IAVs were detected throughout autumn and winter for all sampling locations and years, however, seroprevalence was higher among adults and varied among years. Mallards exhibited individual heterogeneity with regard to immune response, providing instances of both seroconversion and seroreversion to detected viral subtypes. The probability that an individual transitioned from one serostatus to another varied by age, with juvenile mallards having higher rates of seroconversion and seroreversion than adults. Our study provides evidence that a diversity of IAVs circulate in populations of mallards wintering at urban locations in Alaska, and we suggest waterfowl wintering at high-latitudes may play an important role in maintenance of viruses across breeding seasons.

## Introduction

Extensive surveillance sampling for influenza A viruses (IAVs) and IAV antibodies at high-latitude regions such as Alaska throughout summer and autumn has helped to elucidate patterns of viral infection in migratory birds [1–3]. For waterfowl, a major reservoir host of IAVs [4], late-summer/early-autumn is generally considered the period of peak IAV prevalence in dabbling duck (family Anatidae, tribe Anatini) populations, as birds congregate in high densities prior to migration [5, 6]. During this time, a seasonal increase in the proportion of immunologically naïve juvenile birds, and increased bird-to-bird interactions during staging, leads to efficient transmission and dispersal of viruses through migration [7]. However, as migrating waterfowl reach lower-latitude wintering areas, prevalence declines as the proportion of individuals with antibody protection increases [8, 9]. Since most waterfowl species depart from northern autumn staging areas to more southerly staging and wintering areas, surveillance efforts have not focused on waterfowl remaining at high-latitude locations during late autumn and winter. Consequently, limited empirical data exist to assess the potential maintenance of IAVs in biotic (wild birds) or abiotic (waterbird habitats) viral reservoirs in high-latitude regions during the non-breeding season.

Several previous investigations have explored the perpetuation of IAVs in Alaska between breeding seasons; however, the potential for persistence of viruses overwinter is still poorly understood. In an investigation conducted in interior and coastal wetlands of Alaska, Ito et al. [10] isolated IAVs from lake water during late summer/autumn, and therefore speculated that IAVs could potentially be maintained in Alaskan lakes overwinter. Yet, most of the lakes were sampled in late summer/autumn when ducks were present at wetlands and therefore the ability of viruses to remain viable overwinter in Alaskan wetlands was not clearly demonstrated. Lang et al. [11] amplified IAV gene segments from pond sediment samples collected monthly throughout winter at an interior Alaskan wetland. However, despite identifying a diverse array of hemagglutinin (HA) viral subtypes from RNA extracted from sediment samples, the authors were unable to isolate viable IAVs [11]. Using genetic approaches, two investigations [7, 12] identified highly similar IAVs infecting dabbling ducks across years at multiple locations in Alaska, suggesting viral persistence may take place between breeding seasons. However, the relative importance of viral maintenance in Alaska during winter versus the introduction of IAVs by migrating waterfowl arriving in spring remains unclear. Thus, while these studies collectively provide support for potential interannual persistence of IAVs in Alaska, the mechanism(s) maintaining viruses between breeding seasons at high-latitude locations remain unresolved.

Waterfowl wintering in Alaska have only recently been targeted as part of IAV surveillance efforts, despite the potential for viral maintenance in these populations [13]. Ecological alterations, such as warm water discharges and anthropogenic food subsidies provided in two cities of Alaska have created open-water habitats with sufficient resources for birds to overwinter. For example, the number of mallards (*Anas platyrhynchos*) observed during winter in Fairbanks and Anchorage, Alaska, has increased substantially in recent years [14]. Our principal objective was to better understand the potential role of wintering waterfowl in the maintenance of IAVs at high-latitude regions of North America and to gain insights into how population immunity may relate to viral dynamics. We collected swab and serum samples from mallards over the course of three non-breeding seasons at two urban locations in Alaska to quantify: (1) seasonal rates of IAV infection and antigenic diversity of viruses infecting birds, (2) variation in population IAV seroprevalence, (3) the individual immune response of recaptured individuals previously exposed to an IAV, and (4) probabilities of individuals to develop (seroconversion) or lose (seroreversion) detectable antibodies to an IAV during the non-breeding season.

## Materials and methods

### Ethics statement

Capture and processing of wild mallards was approved by the Institutional Animal Care and Use Committee at the University of Alaska Fairbanks (UAF; 358515-11/662280-3) and was authorized by U.S. Federal Bird Banding Permits (#08350 and #23191). All mallards were released at original capture locations after handling.

### Sample locations and years

During August–April of 2012/13 and 2013/14, mallards were captured on the Chena River in Fairbanks, Alaska (64°50’N, 147°45’W). This section of the Chena River remains open throughout winter due to warm water effluent from a local power plant. Approximately 400–600 mallards wintered in this location during the study period [14]. We also captured mallards at Westchester Lagoon (61°12’N, 149°54’W), and Cuddy Midtown Park in Anchorage, Alaska (61°11’N, 149°52’W), from September–April of 2014/15. An estimated 1200–1500 mallards overwintered in Anchorage during our study [14], where they frequently concentrated in small areas of open freshwater on municipal parklands and coastal marsh habitats. Mallards were captured using swim-in bait traps, walk-in bait traps, whoosh nets, and net guns. We determined the sex and age of captured birds based on cloacal and feather examination [15, 16], and marked all birds with U.S. Geological Survey metal leg bands.

### Influenza A virus RNA detection and virus isolation

Cloacal and oropharyngeal swabs were collected from mallards during initial and subsequent captures using sterile polyester tipped swabs. Swabs samples were placed into viral transport media (VTM) (M4RT from Remel Inc., Lenexa, KS, USA) and immediately stored at -80°C (or below) until processing. Viral RNA was extracted from 50 μl of VTM sample using the Omega Mag-Bind Viral DNA/RNA kit (Omega Bio-Tek, Norcross, GA, USA) and a Kingfisher Magnetic Particle Processor (Thermo Scientific, Waltham, MA, USA). RNA was screened using qScript XLT One-Step RT-qPCR ToughMix (Quanta Biosciences, Gaithersburg, MD, USA) and analyzed for fluorescence on an ABI 7500 real-time PCR System (Applied Biosystems, Foster City, CA, USA) for a conserved IAV matrix gene segment (MA) target, as previously described [17]. Samples producing cycle threshold (Ct) values ≤ 45 were considered positive for IAV RNA and individual birds were considered infected if either a cloacal or oropharyngeal swab was determined as being positive for IAV RNA. Positive samples were inoculated into the allantoic cavity of 10 day old embryonated chicken eggs (ECEs) (Charles River, CT, USA), and incubated at 37°C for 72 hours. RNA was extracted from 50 μl of amnio-allantoic fluid (AAF) and screened for the IAV MA gene as described above. Whole genome sequencing was performed on RNA from IAV positive AAF (Ct ≤ 45) at either the Massachusetts Institute of Technology BioMicro Center in Cambridge, MA or the J. Craig Venter Institute in Rockville, MD, as previously described [18]. Each isolate was assigned an HA and neuraminidase (NA) subtype based on the highest percentage identity for respective gene segments using the nucleotide BLAST function on GenBank.

### Serum collection and analysis

We obtained approximately 1.5 ml of whole blood from the jugular vein of mallards during initial capture and on re-capture occasions if birds had not been bled within the previous 7 days. Sera samples were separated through centrifugation and serum was stored at -20°C until analysis. Sera samples were screened for antibodies to the IAV nucleoprotein (NP) gene segment [19, 20] using a commercially available blocking enzyme-linked immunosorbent assay (bELISA; AI MultiS-Screen Avian Influenza Virus Antibody Test Kit; IDEXX Laboratories, Westbrook, Maine, USA) following the manufacturer’s instructions. We considered sample to negative control ratio (S/N) values less than 0.5 as positive based on the manufacturer’s recommendations. While alternative threshold values (0.6–0.7) may increase the sensitivity of the assay [19, 21], this change is accompanied by a slight decrease in specificity, and we prioritized the 100% specificity of this assay for detection of antibodies to IAVs.

We used virus microneutralization (MN) assays to H1 –H12 IAV subtypes to further characterize the individual immune response of mallards found to be seropositive on multiple capture occasions. Antigens for MN assays were prepared in Madin Darby Canine Kidney cells (MDCK; American Type Culture Collection, Manassas VA, USA). During virus propagation, and in all MN assay procedures, cells were maintained in minimal essential media (MEM; Sigma-Aldrich, St. Louis MO, USA) containing TPCK-trypsin (final concentration of 1μg/ml; Worthington Biochemical Corporation, Lakewood, NJ, USA) and antibiotics (final concentration of 100 units penicillin, 0.1mg streptomycin, and 0.25 μg amphotericin B/ml; Sigma-Aldrich). Antigen was stored at -80°C until used. For antibody testing, sera were diluted 1:10 in MEM and heat inactivated at 57°C for 30 minutes. Serum samples were screened at a 1:20 dilution against all antigens. For the screen, 25 μl of the diluted serum (1:10) were placed in a single well of a 96-well v-bottom plate corresponding to each antigen. An additional well for each serum sample served as a serum control to determine potential toxicity. A positive control well using chicken antisera to each antigen (provided by the National Veterinary Services Laboratory, APHIS, USDA) and a negative control well using MEM were also included. Each antigen (25 μl containing 100 median tissue culture infective doses [10^2.0^ TCID_50_]) was added to each well, not including the serum control wells, which received 25 μl MEM. Plates were incubated for 2 hr at room temperature after which 25 μl from each well was transferred to a second 96-well tissue culture plate with a confluent monolayer of MDCK cells. Prior to transfer, the tissue culture plate containing the MDCK cells was washed two times with Dulbecco’s phosphate buffered saline (Sigma-Aldridge) and 150 μl of trypsin supplemented MEM was added to each well. The inoculated tissue culture plate was incubated at 5% CO_2_ at 37°C and was visually read at 72 hours. For the test result to be considered valid, all controls (serum, positive, and negative) had to meet their expected negative or positive status. In addition, based on back titration in MDCK cells (four replicates per dilution), the viral titer of the antigen had to fall within 10^1.5^ and 10^2.5^ TCID_50_/25 μl. Sera were considered positive on the screen if no cytopathic effect (CPE) was observed. All positive serum samples were titrated. Each positive serum sample was diluted two-fold in MEM on a 96 well v-bottom plate (final volume of 25 μl. well at dilutions 1:20 to 1:640) and tested as described above. If CPE was observed at the minimum 1:20 dilution, the sample was classified as negative; if not, the positive titer was recorded as the highest dilution at which no CPE was observed. Viruses used as antigens in the MN assays included A/mallard/MN/AI12-4297/2012 (H1N1), A/mallard/MN/AI08-2755/2008 (H2N3), A/mallard/MN/AI10-2593/2010 (H3N8), A/mallard/MN/AI10-3208/2010 (H4N6), A/mallard/MN/AI11-3933/2011 (H5N1), A/mallard/MN/AI08-2721/2008 (H6N1), A/mallard/MN/AI08-3770/2009 (H7N9), A/mallard/MN/SG-01048/2008 (H8N4), A/RUTU/DE/AI11-809/2011 (H9N2), A/mallard/MN/SG-00999/2008 (H10N7), A/mallard/MN/SG-00930/2008 (H11N9), and A/mallard/MN/SG-3285/2007 (H12N5).

### Statistical analyses

We used generalized linear logistic regression models and an information theoretic approach to assess patterns of variation in IAV prevalence and seroprevalence. We considered variation relative to month and season, with seasons defined as: autumn (August–October), early-winter (November–January), and late-winter (February–April). In each analysis, we randomly selected one capture occasion from each season for individuals captured on multiple occasions. To assess differences in IAV infection status and seroprevalence associated with host age, we defined two distinct age classes for mallards, with birds characterized as being either juvenile (hatched the previous summer; HY) or adult (> 1 year old; AHY). Our candidate model sets assessing sources of variation in IAV prevalence and seroprevalence consisted of 56 models that contained various additive and multiplicative effects of the variables age, sex, monthly trend, season, and year (Table A, C in S1 File). Model support was evaluated using Akaike’s Information Criterion corrected for sample size (AICc) [22]. We eliminated models with equivalent structure and one additional parameter from consideration and in the case of model selection uncertainty, we present model averaged estimates from supported models within 4 ΔAIC of the top approximating model [23].

We used multi-state models in Program MARK [24, 25] to estimate the probability of seroconversion or seroreversion to IAVs for mallards sampled in Anchorage (the location with the largest sample size). This analysis included capture histories of the 82 individuals captured on at least 2 distinct occasions. We estimated state transition probabilities (Ψ_i_) and capture probability (*p*_*i*_), using a two stage approach. In the first stage, we constrained Ψ to a highly parameterized structure and considered 4 models explaining variation in *p*; these models allowed *p* to vary relative to age, sex, and a monthly trend. In the second stage, we fixed *p* to the top supported structure from stage 1 and considered 4 models to assess competing hypotheses regarding sources of variation in Ψ. Survival was held constant in all models. Estimates were back-transformed from the logit link and are presented ± SE unless otherwise specified.

To gain inference on trends in antibody titers through time, we plotted the change in titer for antibodies to H1 –H12 HA subtype viruses for recaptured individuals inferred as being seropositive on two or more occasions. We randomly selected two capture occasions to assess subtype-specific antibody changes for seropositive individuals with more than two captures during the 2014–2015 sampling year. We plotted trendlines for specific HA subtype antibodies if detections occurred in multiple months across the study period and considered antibody titers changing ≤ 2 log titers as “stable” and titers changing by > 2 log titers as either increasing or decreasing. Associated coefficient of determination (*R*^2^) values and (Δlog) titer values were estimated using the R environment [26].

## Results

### IAV prevalence & subtype diversity

We detected IAV RNA in 134 of 1182 cloacal swab samples and 67 of 1186 oropharyngeal swabs collected from 923 mallards captured in Fairbanks and Anchorage over the course of our study. After reducing our dataset to samples from initial captures and one randomly selected recapture event in other seasons, our summary of IAV prevalence included 1062 paired swab samples from 963 capture occasions with an apparent prevalence of 17% (Table 1). In our analysis of IAV prevalence, the top AICc approximating model (*w* = 0.43) allowed prevalence to vary by age, season, year, and the interaction between season and year (Table 2, Table A in S1 File). A model with the same variables, but containing an age*season interaction term, also received substantial support (*w* = 0.17; Table 2, Table A in S1 File); we present model averaged predictions from these two models.

**Table 1:** Summary of mallard captures and recapture screening results for molecular detection of viral RNA by rRT-PCR and influenza A virus antibodies using a bELISA.

Year	No. captures	No. recaptures	Positive M rRT-PCR birds (%)^a^	Viruses isolated (%)^a^	Seropositive birds (%)^a^
2012/13	277	43	54/308(18)	7/308(2)	107/250(43)
2013/14	162	13	22/167(13)	8/167(5)	57/163(35)
2014/15	484	216	105/587(18)	48/587(8)	293/571(51)
Total	923	272	181/1062(17)	63/1062(6)	457/984(46)

^a^ Sample sizes, number of positive samples, and estimates from original captures and one randomly selected occasion from each season for birds captured on multiple occasions.

**Table 2:** Results from GLM logistic regression models predicting variation in influenza A virus prevalence for mallards sampled in Fairbanks and Anchorage, Alaska from August through April of 2012–2015.

Model Name	*K*^a^	AICc^b^	Δ AICc	*w*_i_^b^
Age+Season*Year	10	913.65	0.00	0.43
Age+Sex+Season*Year	11	915.15	1.51	0.20
Age*Season*Year	18	915.48	1.83	0.17
Age*Sex+Season*Year	12	915.54	1.90	0.17
Age*C.Month*Year	12	919.34	5.69	0.02

^a^ Number of parameters in the model.

^b^Akaike’s Information Criterion corrected for sample size (AICc) and model weight (*w*_i_) relative to others in the candidate model set.

**Fig 1:**
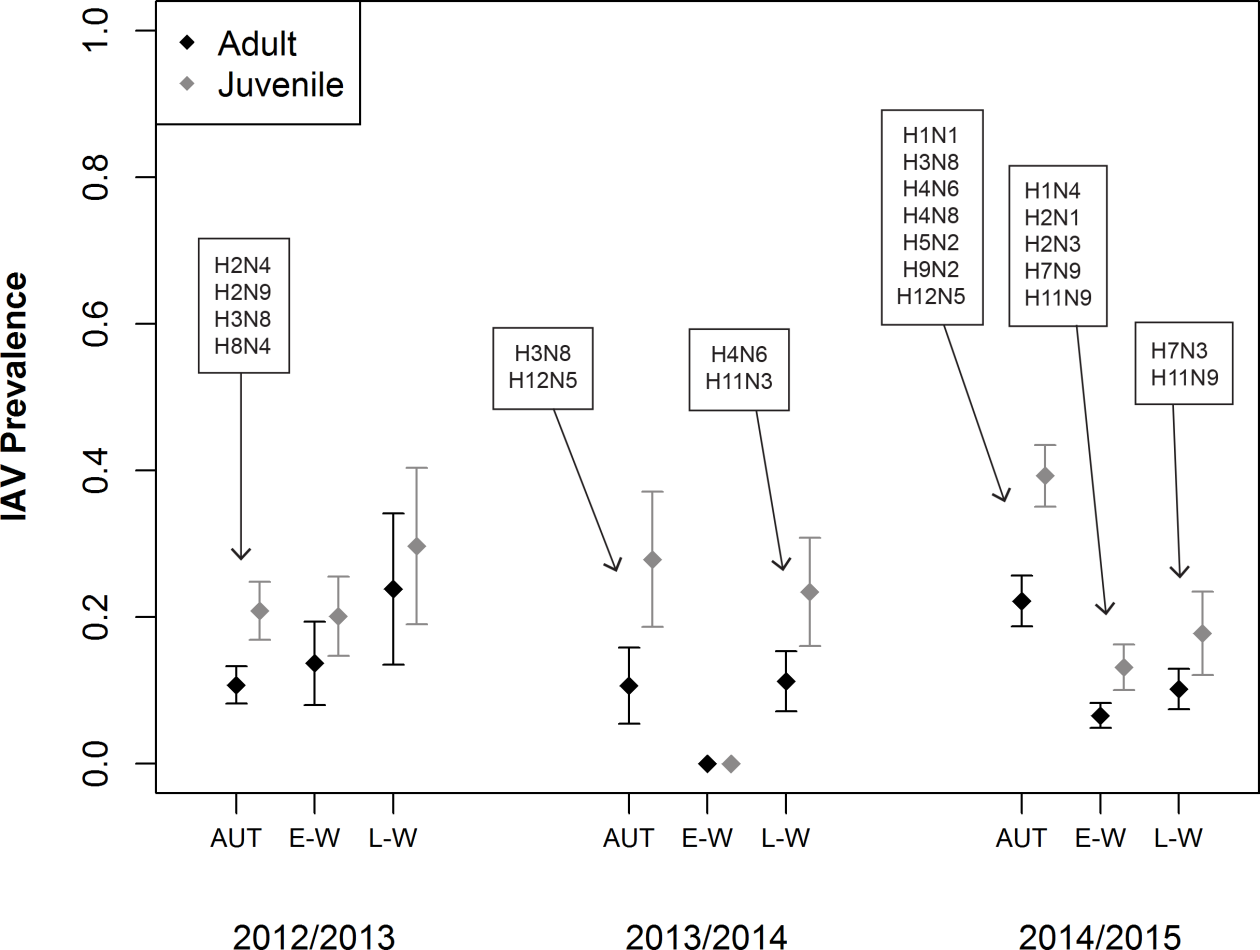
Estimated prevalence (± SE) of influenza A virus (IAV). Mallards were captured and sampled in Fairbanks and Anchorage, Alaska during August–April of 2012–2015. Estimates represent prevalence in autumn (AUT; Aug–Oct), early-winter (E-W; Nov–Jan), and late-winter (L-W; Feb–Apr). Boxes indicate viral subtype combinations isolated during a given season.

Estimated IAV prevalence was higher in juveniles than adults, especially during the autumn season (Fig 1; Table B in S1 File). During 2012/13 in Fairbanks, estimated IAV prevalence remained relatively stable between autumn and late-winter (Fig 1). Prevalence estimates for this sampling location/year were lower for both age classes during autumn (AHY: 0.11 ± 0.03, HY: 0.21 ± 0.04) as compared to late-winter (AHY: 0.24 ± 0.10, HY: 0.30 ± 0.11). During the 2013/14 field season in Fairbanks, estimated IAV prevalence for both age classes was similar in autumn (AHY: 0.13 ± 0.04, HY: 0.24 ± 0.06) and late-winter (AHY: 0.12 ± 0.04, HY: 0.22 ± 0.06) but we did not detect any positive samples from the 74 birds sampled during early-winter (Fig 1). In Anchorage during the 2014/15 sampling year, IAV prevalence declined from autumn to early- and late-winter (Fig 1). Prevalence was higher for juveniles (HY: 0.39 ± 0.04) than adults (Fig 1; AHY: 0.22 ± 0.03) during autumn, but this difference was less pronounced during early-winter (AHY: 0.07 ± 0.02, HY: 0.13 ± 0.03) and late-winter (AHY: 0.10 ± 0.03, HY: 0.18 ± 0.06).

Virus isolation yielded isolates from 6% of the 1062 mallards included in the analysis of IAV prevalence, with isolates from 3% of mallards in 2012/13, 5% of mallards from 2013/14, and 8% of mallards during 2014/15 (Table 1). Across sampling years, only H3N8 was isolated in every year of the study and H4N6 and H12N5 were isolated from mallards in Fairbanks during 2013/14 and Anchorage in 2014/15 (Fig 1). In Fairbanks, additional IAV subtypes isolated included H2N4, H2N9, and H8N4 during 2012/13 and H11N3 during 2013/14 (Fig 1). In Anchorage during 2014/15, additional IAV subtypes isolated included H1N1, H2N1, H2N3, H5N2, H7N3, H9N2, and H11N9 (Fig 1).

### Antibody prevalence

We detected antibodies to IAVs in 495 of 1061 serum samples from mallards in Fairbanks and Anchorage collected over three successive sampling years. After reducing our dataset to samples from initial captures and a randomly selected recapture event in other seasons, our summary of IAV seroprevalence included 984 serum samples from 897 individuals, yielding an apparent seroprevalence of 46%. Antibodies to IAV were detected in 43% of the samples from 2012/13, 35% of the samples from 2013/14, and 51% of samples from 2014/15 (Table 1).

**Fig 2:**
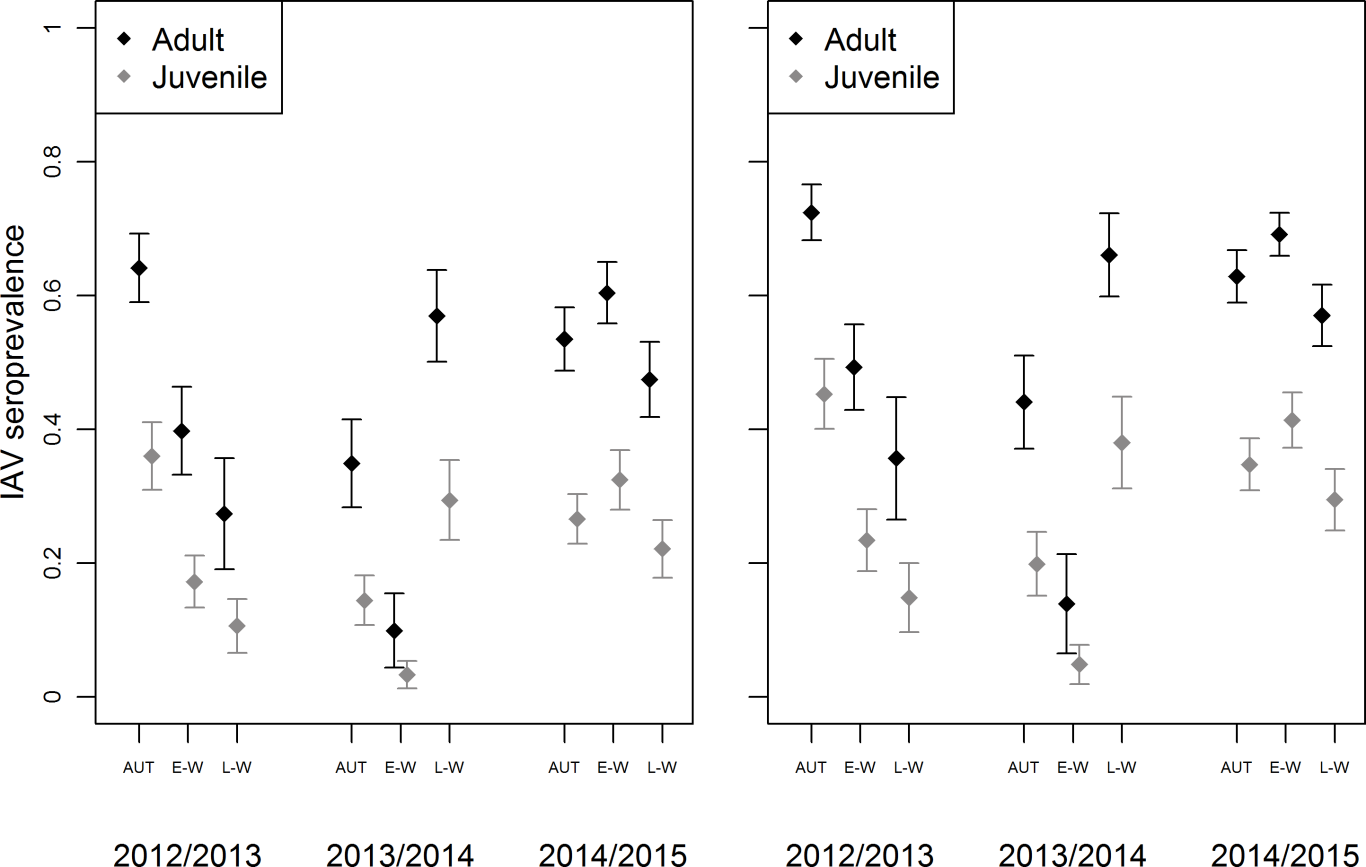
Estimated influenza A virus (IAV) seroprevalence (± SE) for female (left) and male (right) mallards. Mallards were captured and sampled in Fairbanks and Anchorage, Alaska during August–April of 2012–2015. Estimates represent seroprevalence in autumn (AUT; Aug–Oct), early-winter (E-W; Nov–Jan), and late-winter (L-W; Feb–Apr).

Our top AICc approximating model (*w* = 0.67) indicated support for variation in seroprevalence relative to age, sex, season, year, and the interaction between season and year (Table 3, Table C in S1 File). Seroprevalence estimates were higher for adults than juveniles and higher for males than females (Fig 2; Table D in S1 File). Across seasons and years, estimates of IAV seroprevalence were highest in adults (M: 0.72 ± 0.04, F: 0.64 ± 0.05) during autumn of 2012/13 and lowest in juveniles (M: 0.05 ± 0.03, F: 0.03 ± 0.02) during early-winter of 2013/14. During 2012/13 in Fairbanks, IAV seroprevalence declined through late-winter in both adult (M: 0.36 ± 0.09, F: 0.27 ± 0.08) and juvenile (M: 0.36 ± 0.09, F: 0.27 ± 0.08) mallards (Fig 2). During 2013/14 in Fairbanks, IAV seroprevalence estimates were higher in both autumn (M: 0.20 ± 0.05, F: 0.14 ± 0.04) and late-winter (M: 0.38 ± 0.07, F: 0.29 ± 0.06) as compared to early-winter (Fig 2). During 2014/15 in Anchorage, estimates of IAV seroprevalence remained higher and more stable across all seasons than during two autumn/winters in Fairbanks (Fig 2).

**Table 3:** Logistic regression models predicting variation in influenza A virus seroprevalence for mallards sampled in Fairbanks and Anchorage, Alaska from August through April of 2012–2015.

Model Name	*K*^a^	AICc^b^	Δ AICc	*w*_i_^b^
Age+Sex+Season*Year	11	1244.46	0.00	0.67
Age*Sex+Season*Year	12	1246.49	2.03	0.24
Age+Season*Year	10	1248.83	4.37	0.07
Age+Sex*Season*Year	19	1252.03	7.57	0.02

^a^ Number of parameters in the model.

^b^Akaike’s Information Criterion corrected for sample size (AICc) and model weight (*w*_i_) relative to others in the candidate model set.

### Serostatus state transitions

Of the 82 mallards recaptured in Anchorage, 63 individuals maintained the same serostatus, whereas 19 individuals transitioned from one serostatus to the other (seroconversion = 8, seroreversion = 11; Table 4). The top AICc approximating model indicated support for variation in capture probability by a monthly trend; the probability of state transition varied relative to age (Table E in S1 File). The probabilities of seroconversion (0.19 ± 0.07) and seroreversion (0.54 ± 0.14) for juveniles were considerably higher than for adults (seroconversion = 0.05 ± 0.03, seroreversion = 0.05 ± 0.03; Table 5).

**Table 4:** Serostatus histories for 82 mallards captured on multiple occasions during the winter of 2014/15 in Anchorage, Alaska. Serostatus of juvenile (HY) and adult (AHY) mallards was determined to be either negative (-) or positive (+) through bELISA.

Group	(n)	(-)→(-)	(+)→(+)	(-)→(+)	(+)→(-)
AHY	43	15	24	2	2
HY	39	18	6	6	9
Total	82	33	30	8	11

**Table 5:** Estimates of serostatus transition probability (Ψi) and capture probability ($\hat{p}$) from the top approximating model for adult (AHY) and juvenile (HY) mallards captured in Anchorage, Alaska from September through April (2014/15). Seroconversion (-)→(+) and seroreversion (+)→(-) estimates are representative of each age class.

Age	Ψ_i_ = (-)→(+)	SE	Ψ_i_ = (+)→(-)	SE	Month	$\hat{p}$	SE
AHY	0.05	0.03	0.05	0.03	Oct	0.46	0.06
					Nov	0.39	0.04
					Dec	0.32	0.03
					Jan	0.26	0.02
					Feb	0.21	0.02
					Mar	0.16	0.02
					Apr	0.12	0.02
HY	0.19	0.07	0.54	0.14	Oct	0.46	0.06
					Nov	0.39	0.04
					Dec	0.32	0.03
					Jan	0.26	0.02
					Feb	0.21	0.02
					Mar	0.16	0.02
					Apr	0.12	0.02

### Virus MN assay

**Fig 3:**
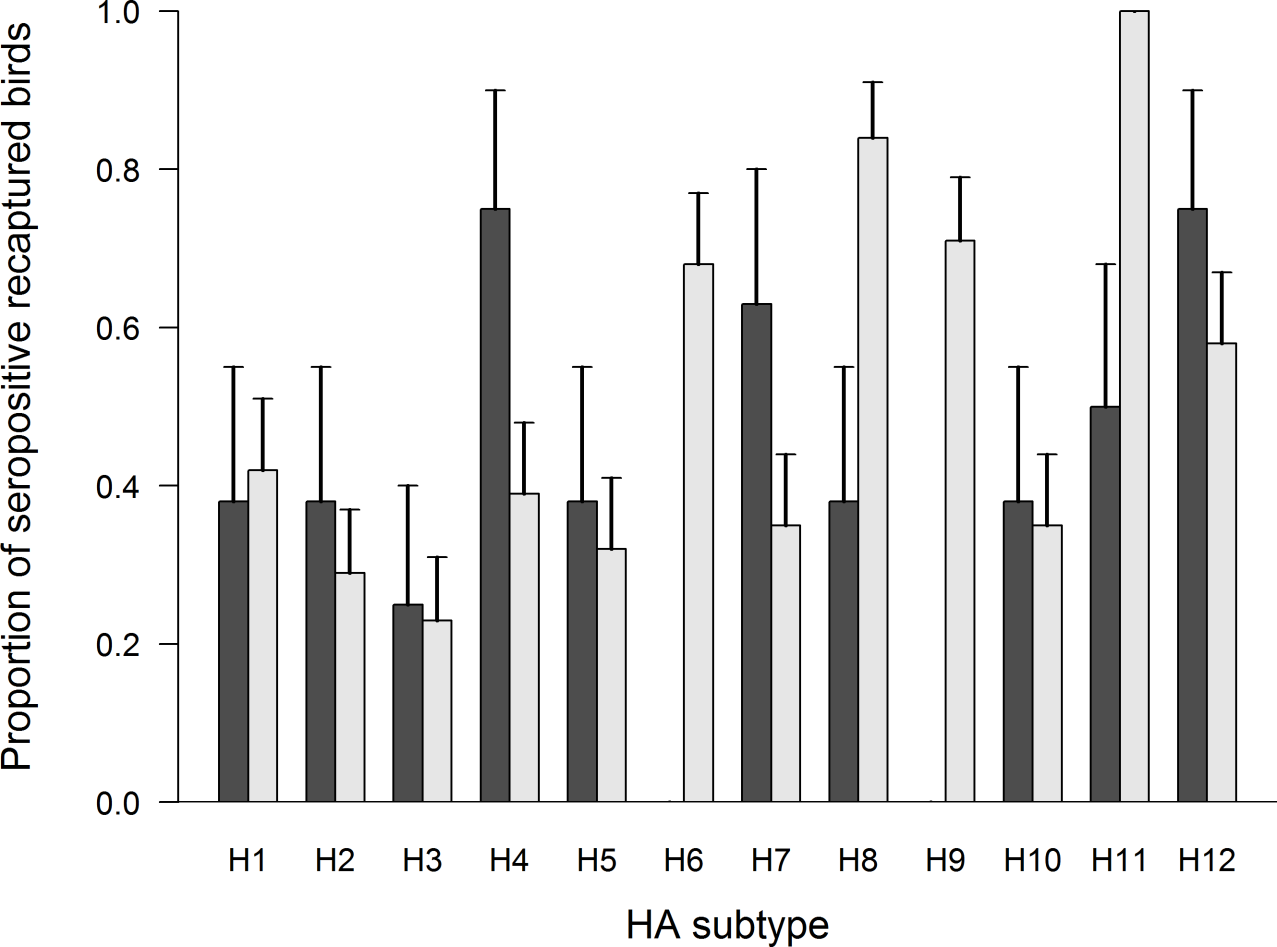
Subtype-specific antibody diversity to H1-H12 HA influenza A viruses (IAVs) in mallards. Samples were collected from mallards determined to be seropositive on multiple capture occasions from August–April of 2012/13 in Fairbanks (dark gray bars) and from mallards captured from September–April of 2014/15 in Anchorage (light gray bars).

**Fig 4:**
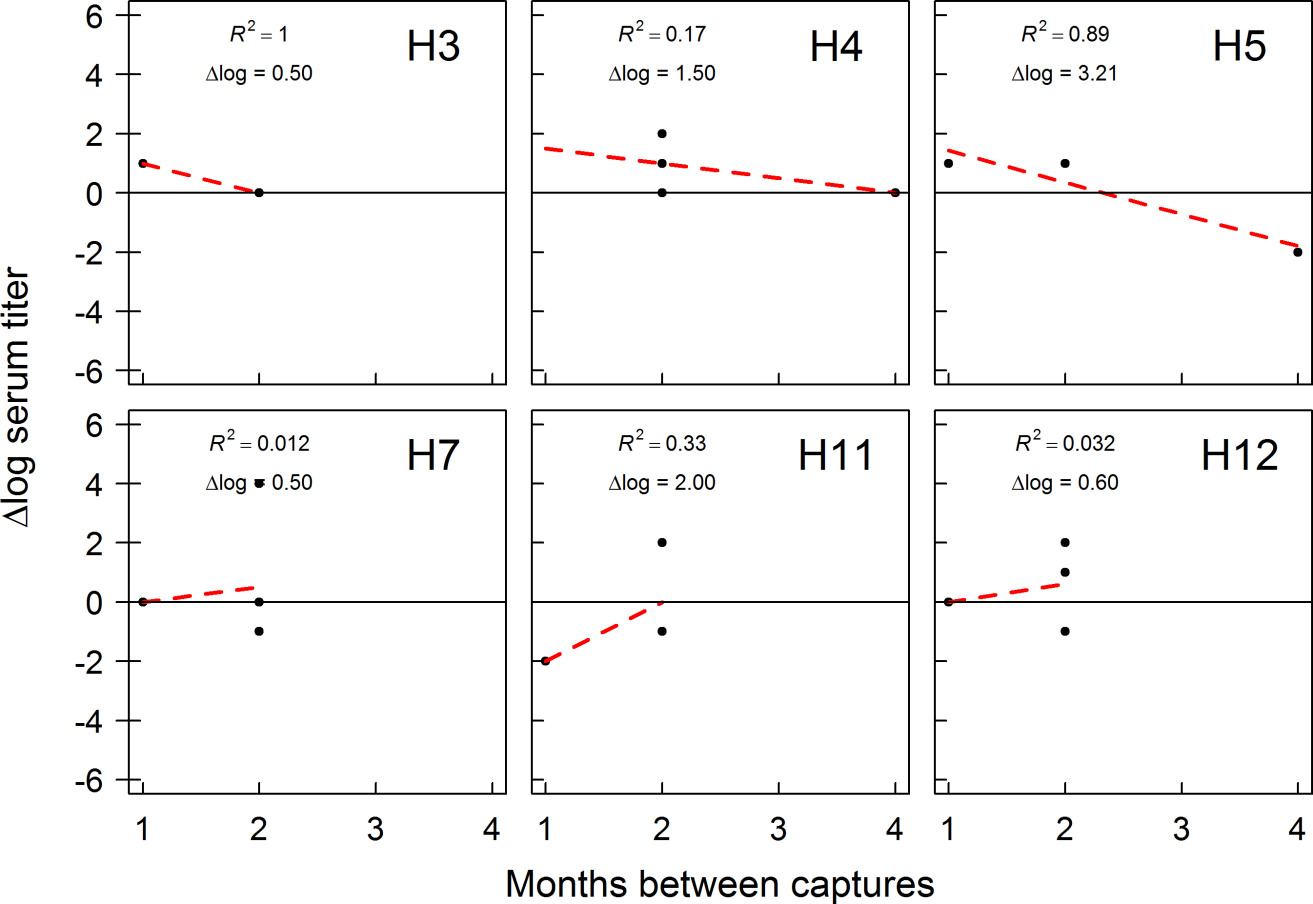
Trends in antibody production to influenza A viruses (IAVs) by mallards sampled in Fairbanks, Alaska. Mallards were captured and sampled from August 2012 through April of 2013. Analysis included samples obtained from individuals determined to be seropositive on multiple capture occasions with bELISA. Time intervals on the x-axis represent the number of months between first and one randomly selected capture occasion. The dashed red line represents a linear regression trend line and associated R-squared value.

**Fig 5:**
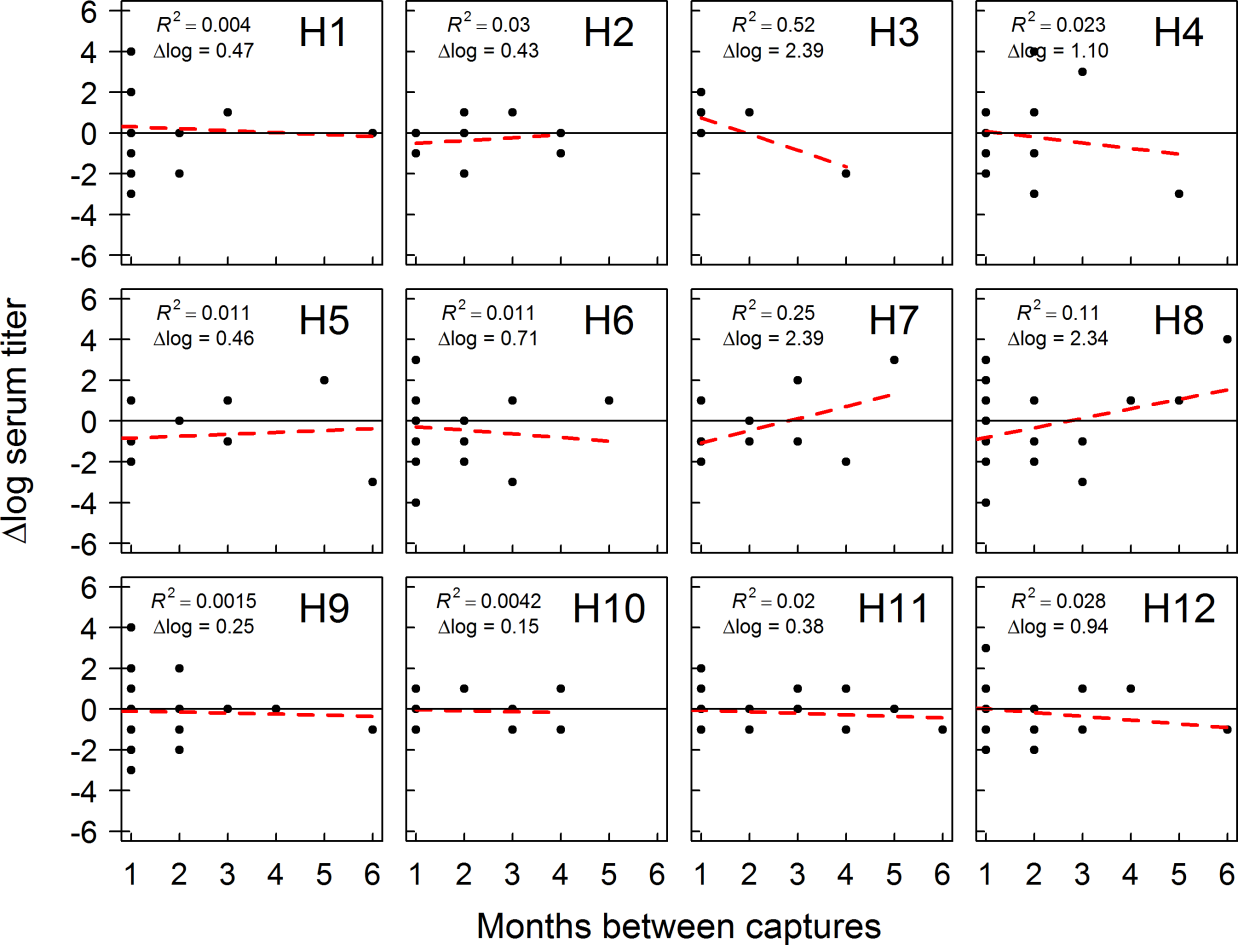
Trends in antibody production to influenza A viruses (IAVs) by mallards sampled in Anchorage, Alaska. Mallards were captured and sampled from September through April of 2014/15. Analysis included samples obtained from individuals determined to be seropositive upon multiple capture occasions with bELISA. Time intervals on the x-axis represent the number of months between first and one randomly selected capture occasion. The dashed red line represents a linear regression trend line and associated R-squared value.

Of 284 birds recaptured over the course of our study, 39 were seropositive on at least two capture occasions within the same year. Eight of these individuals were sampled in Fairbanks during 2012/13 (Table F in S1 File) and 31 individuals were sampled in Anchorage during 2014/15 (Table G in S1 File). The single individual recaptured during 2013/14 in Fairbanks was excluded from analysis. We identified antibody titers to some (10/12) H1 –H12 HA subtype viruses in recaptured mallards sampled in Fairbanks in 2012/13 and all H1 –H12 HA subtype viruses in recaptured mallards sampled in Anchorage during 2014/15 (Fig 3). For recaptured mallards sampled in Fairbanks in 2012/13, we had sufficient data to assess trends in antibody titers for six HA viral subtypes (Fig 4). Antibody titers to H5 subtype IAVs appeared to decrease between capture occasions, while antibody titers to other IAV subtypes (H3, H4, H7, H11, H12) were inferred to be stable (≤ 2 log titer change) through time. A larger number of recaptured mallards in Anchorage in 2014/15 allowed us to assess trends in antibody titers to 12 HA subtype viruses (Fig 5). For Anchorage mallards, antibody titers appeared to remain stable to the majority (9/12) of H1–12 HA viral subtypes between capture occasions. The three exceptions were an apparent decline in antibody titers to H3 HA subtype viruses and increases in antibody titers to H7 and H8 HA subtype viruses over the course of the 2014/15 sampling period (Fig 5).

## Discussion

### Viral prevalence

In this study, we present information on the dynamics of IAVs within mallard populations at the northern extent of their winter distribution, providing evidence for perpetuation of IAVs throughout the non-breeding period. While our estimates of IAV prevalence for mallards during autumn (Aug–Oct) are similar to those reported by previous studies of dabbling ducks in occurring during autumn in Alaska [2, 27, 28], we also provide evidence for putatively high rates of IAV infection throughout early-winter (Nov–Jan) and late-winter (Feb–Apr). Thus, waterfowl wintering at high-latitudes may be infected with IAVs in late-winter (Feb–Apr) at levels similar to or perhaps even higher than reported in previous studies of North American and European waterfowl during winter at lower latitudes [29–32].

In agreement with previous studies investigating IAV dynamics in post-breeding waterfowl [5, 6], our results indicate that prevalence during autumn was higher for juvenile mallards than adults, which is congruent with susceptibility of immunologically naïve juvenile ducks to IAVs post-fledging [33]. The effect of age remained throughout the non-breeding season, although age-specific differences in prevalence estimates were less pronounced during early-(Nov–Jan) and late-winter (Feb–Apr) than during autumn (Aug–Oct). This suggests that IAV prevalence approaches equivalence among waterfowl age classes through time which may be a function of adaptive immune responses of juvenile birds more closely resembling those of adults as they are repeatedly exposed to locally circulating IAVs throughout the non-breeding period [34].

Site-specific prevalence estimates were comparable, but we isolated a broader diversity of IAV subtypes in Anchorage, where our rate of virus isolation was higher as compared to Fairbanks. This may be a function of differences in the populations in which IAVs are maintained or environmental conditions facilitating persistence of IAVs throughout the winter period. In Fairbanks, 400–600 mallards used a small (~2 km) section of open water on the Chena River, and tended to concentrate at the location where supplemental feed was provided daily. In Anchorage, a larger number of mallards (1200–1500) used a number of small ponds and several riverine locations over a much broader area, and birds were frequently observed traveling between locations throughout the day. The larger population of mallards in Anchorage combined with a more variable distribution of individuals among habitats may have supported a greater diversity of viruses. Furthermore, the proximity to marine habitats and direct connectivity to other areas with wintering waterfowl populations may have increased the likelihood for introduction of viruses in the Anchorage population. Alternatively, differences in environmental conditions may have contributed to the seasonal/geographic variation in virus isolation results. Because we did not record variables such as water temperature, pH, and salinity, which may affect the perpetuation of IAVs in aquatic reservoirs [35–37], our strength of inference regarding the role of environmental factors in perpetuation and transmission of IAVs at the Chena River in Fairbanks and capture locations in Anchorage is limited.

### Antibody prevalence

Seroprevalence was consistently elevated during autumn (Aug–Nov) in comparison to other seasons (early- & late-winter) across all three years of the study. Additionally, seasonal variation was more pronounced in Fairbanks than in Anchorage and geographic variation in seroprevalence may reflect location-specific differences in viral dynamics between the two sample populations. For example, our estimates of IAV prevalence in mallards were lower in both years in Fairbanks during autumn than for mallards in Anchorage. Thus, we might expect that population immunity in Fairbanks birds following the presumptive peak in prevalence would also be lower during the early-winter and late-winter seasons. Alternatively, we may have missed the peak in IAV prevalence during our discrete sampling efforts, resulting in the mismatch between prevalence and seroprevalence patterns during both years in Fairbanks. Consequently, IAV seroprevalence did not exhibit an expected increase following autumn peaks in viral prevalence in Fairbanks, whereas seroprevalence remained high in both early and late-winter during 2014/15 in Anchorage.

Consistent with results from previous investigations of IAV seroprevalence in waterfowl [3, 38, 39], our estimates of seroprevalence were higher for adults than juveniles. Increased probability of previous IAV exposure in adults, likely resulting in a long-lasting immune response, presumably contributed to our observed age-specific variation in seroprevalence [40–42]. We also found support for variation in seroprevalence by sex, with higher estimates for males than females. This finding corresponds with sex-specific differences in IAV prevalence reported for dabbling ducks sampled during late summer at a nearby interior Alaska breeding location [28], but differs from results of several studies that reported higher rates of IAV seroprevalence in female than male waterfowl [3, 38, 39]. Such discrepancies across studies may be due to seasonal, geographic, or species differences in sex-specific IAV exposure or immune response. Ultimately, sex-specific seroprevalence patterns in dabbling ducks may be influenced by life-history characteristics of the population sampled. For example, during winter, females must acquire sufficient nutrient stores in preparation for each potential breeding effort. Despite consistent availability of anthropogenic foods at our capture locations, which may potentially afford more energy for immune responses to circulating IAVs [43], lower seroprevalence estimates of females in our study may reflect a trade-off between increased immunity and increased body condition required for the upcoming breeding season.

### Probability of seroconversion/reversion

We found support for age-specific differences in the probabilities of seroconversion and seroreversion, with juveniles having higher probabilities for both rates than adults. Because most recaptured individuals maintained their serostatus across capture occasions, the elevated rates of seroconversion/reversion for juvenile mallards suggests age-specific differences in the duration of antibody production and transition of B cells to a memory state. This finding supports results from captive studies suggesting that immunologically naïve juvenile mallards exhibit a shorter- immune response period than adults previously exposed to a similar antigen [44]. Furthermore, low estimates of seroconversion/seroreversion rates for adults, suggests that antibody responses in mature birds may be relatively long-lived or that these birds may continually be exposed to IAV antigens. While sample size restrictions limited our ability to test for geographic and sex variation relative to seroconversion/reversion rates, our age-specific estimates for seroconversion and seroreversion are lower in comparison to most within-year estimates reported for other species of waterfowl such as pink-footed geese (*Anser brachyrhynchos*) [38] and lesser snow geese (*Chen caerulescens*) [39]. As IAV dynamics have been shown to vary considerably among tribes of waterfowl [8], discrepancies in our rates of seroconversion/reversion may be attributed to specific differences in IAV dynamics within reservoir host species [8, 45, 46].

### Trends in antibody titers

Mallards sampled in Fairbanks and Anchorage yielded wide subtype-specific antibody diversity. We identified changes in antibody titers to H1 –H12 HA subtype viruses through MN between capture occasions, however we only identified four subtypes (H3, H5, H7, H8) which exhibited a directional trend, suggesting potential variation in the timing of epidemiological peaks of infection for various HA subtypes circulating in our sample populations. In Fairbanks in 2012/13, we found evidence of declining antibody titers to H5 subtype IAVs; however, we did not isolate IAVs of this subtype during our sampling period. It is possible that H5 viruses were circulating among birds during our period of sampling but were undetected or that H5 infections occurred prior to initiation of our sampling efforts in August. Alternatively, seroreactivity observed to H5 subtype IAVs may have represented heterosubtypic immune responses to several closely related antigens (H2N4, H2N9) isolated from Fairbanks mallards during autumn sampling [41, 42, 47].

For Anchorage mallards sampled in 2014/15, we detected declining antibody titers to H3 subtype IAVs, which appears to be consistent with the timing of circulation for H3 HA subtype viruses in the Anchorage population. That is, isolation of H3 subtype viruses occurred only during autumn, and therefore declining titers to H3 HA subtypes is consistent with seroreversion in birds after the epidemiological peak of infection in our study population. Additionally, we identified increasing antibody titers to H7 and H8 subtype viruses. Although we did not detect any H8 subtype viruses, we did isolate H7 subtype viruses during early- and late-winter. Thus, our data comparing trends in antibody titers in wintering populations of mallards relative to results of virus isolation suggests that antibody profiling of wild birds has utility as a supplement to traditional viral sampling by providing inference on the circulation of viral subtypes that may be undetected or under-represented through periodic sampling regimes.

### Conclusions

Our study demonstrates that low-pathogenic IAVs circulate within populations of waterfowl wintering at urban locations in Alaska. Therefore, it is possible that resident waterfowl play a role in IAV maintenance during the non-breeding season at high-latitude locations in North America. As the influx of spring migrating dabbling ducks typically occurs in late-April and May at high-latitude locations, our putatively high estimates of late-winter (Feb–Apr) IAV prevalence suggests transmission among individuals in our sample populations may continue in light of increasing population immunity. Hence, mallards wintering at urban locations in Alaska may serve as a reservoir of IAVs at high-latitude locations, supplementing viruses seeded by spring-migrating waterfowl. Additionally, our study demonstrates the utility of including subtype-specific antibody information for identifying viral subtypes not identified through virus isolation during discrete sampling periods. Inclusion of MN data may provide wildlife managers and regulatory agencies with a more informed snapshot of recently circulating viruses if limited surveillance funding prohibits continuous sampling.

## Supporting information

S1 File Model selection results and coefficients for IAV prevalence and seroprevalence, as well as results of serostatus state transition analyses and virus microneutralization assays. Tables A and B provide the model selection results and estimates for the analysis of IAV prevalence. Tables C and D provide the model selection results and estimates for the analysis of IAV seroprevalence. Table E provides model selection results for the analysis of serostatus state transitions. Tables F and G provide results from virus microneutralization assays of samples collected in Fairbanks (FAI) and Anchorage (ANC).
(XLSX)
